# An extensive dataset for successful recognition of fresh and rotten fruits

**DOI:** 10.1016/j.dib.2022.108552

**Published:** 2022-08-24

**Authors:** Nusrat Sultana, Musfika Jahan, Mohammad Shorif Uddin

**Affiliations:** Department of Computer Science and Engineering, Jahangirnagar University, Dhaka, Bangladesh

**Keywords:** Image classification, Agriculture, Fruit dataset, Deep learning, Computer vision

## Abstract

Detection of rotten fruits is very crucial for agricultural productions and fruit processing as well as packaging industries. Usually, the detection of fresh and rotten fruits is done manually which is an ineffective and lengthy process for farmers. For this reason, the development of a new classification model is required which will reduce human effort, cost, and production time in the agriculture industry by recognizing defects in the fruits. This article offers a major dataset to the researchers to develop effective algorithms for recognizing more variety of fruits and overcome the limitations by increasing accuracy as well as decreasing computation time. This dataset contains sixteen types of fruit classes, namely fresh grape, rotten grape, fresh guava, rotten guava, fresh jujube, rotten jujube, fresh pomegranate, rotten pomegranate, fresh strawberry, rotten strawberry, fresh apple, rotten apple, fresh banana, rotten banana, fresh orange, rotten orange. We collected various fresh and rotten fruit images from 16th to 31st March 2022 from different fruit shops and real fields with the help of a domain specialist from an agricultural organization.

The dataset is hosted by the Department of Computer Science and Engineering, Jahangirnagar University, and is freely available at https://data.mendeley.com/datasets/bdd69gyhv8/1

**Specification Table**

**Table:** 

Subject	Computer Science
Specific subject area	Image Classification, Image Recognition, Deep Learning, and computer vision.
Type of Data	Images
How the data were acquired	From 16th to 31st March 2022, we collected various varieties of fresh and rotten fruit images from different fruit shops and real fields using a Single-lens reflex digital camera (Nikon D5600) to get raw fruit images.
Data format	Raw jpg
Parameters for data collection	Fresh and rotten images of different fruits were collected separately. There exist various types of fruit images including grape, guava, jujube, pomegranate, apple, banana, orange, and strawberry.
Description of Data Collection	No pre-treatment of the samples was done in any research work before. With the assistance of a domain specialist from an agricultural organization, we collected all the fruit images.
Data source location	*Location:* Different fruit shops and real field *Zone:* Savar, Dhaka *Country:* Bangladesh
Data Availability	*Repository name:* Mendeley Data *Data identification number (DOI number):*10.17632/bdd69gyhv8.1 *Link of the dataset:*https://data.mendeley.com/datasets/bdd69gyhv8/1

## Value of the Data

• Classification of fresh and rotten fruits is usually carried out by people, which is ineffective for fruit farmers as well as fruit sellers. For this reason, the development of a new classification model is required which will reduce human effort, cost, and production time in the agriculture industry by recognizing defects in the fruits. To build a better classification model, an efficient dataset is needed.
• This dataset gives a visual representation of fresh and rotten fruits in a hyperspectral order of images. Therefore, it empowers researchers to recognize and classify fresh and rotten fruits at an early stage.
• Gathered Images can be utilized to construct, train, test, analyze, and compare various deep learning algorithms for recognizing fresh and rotten fruits based on different features.
• Original fresh and rotten images are captured from a wider perspective. This dataset contains a larger number of fruit classes compared to other existing datasets [1], [2], [3]. This will help researchers to detect large number of fruit classes and get better performance.
• This data was captured from different fruit shops and real fields in natural weather with inhomogeneous lighting conditions. As a result, researchers may find it difficult to detect exceptions with the naked eye.
• Early recognition of fresh and rotten fruits based on research findings may enable farmers to produce large quantities of fruit and put on value to the national economy.

## 1 Dataset Description

Fruits play a vital role in any country's economic development. Everyone wants to buy fresh, high-quality fruits. Since fruits decay with time, it may have a bad effect on the economy. Approximately one-third of the fruits are projected to be rotten, resulting in significant financial loss. Furthermore, customers believe that damaged fruits are dangerous to their health, and the sale of fruits will be affected. This dataset can be a state-of-the-art mentor in creating algorithms for the early detection and classification of fresh and rotten fruits in the agricultural field in these situations [1]. To develop computer vision-based algorithms, an extensive fruit dataset is presented containing sixteen types of fruit classes, namely fresh apple, rotten apple, fresh banana, rotten banana, fresh orange, rotten orange, fresh grape, rotten grape, fresh guava, rotten guava, fresh jujube, rotten jujube, fresh pomegranate, rotten pomegranate, fresh strawberry, and rotten strawberry. This dataset has a total number of three thousand two hundred (3200) original images and 12,335 augmented images. Our initial training dataset contains 2560 images. We took the pictures using a digital camera with the assistance of a domain expert from an agricultural organization. All the images hold a constant width and height of 512 × 512 pixels.

Table 1 contains details of the dataset and Table 2 contains brief details of the dataset for each fruit class.

**Table 1:** Details of the dataset.

Category	No. of Original Images	No. of Augmented Images
Fresh Apple	200	734
Rotten Apple	200	738
Fresh Banana	200	740
Rotten Banana	200	736
Fresh Orange	200	796
Rotten Orange	200	796
Fresh Grape	200	800
Rotten Grape	200	746
Fresh Guava	200	797
Rotten Guava	200	797
Fresh Jujube	200	793
Rotten Jujube	200	793
Fresh Pomegranate	200	797
Rotten Pomegranate	200	798
Fresh Strawberry	200	737
Rotten Strawberry	200	737
*Total*	3200	12,335

**Table 2:** Brief Details of fruit dataset.

Class Name	Description	Visualization
Fresh Grape	Fresh grape can be identified through its colors, textures and condition of stems. The grapes should be firm, plump, and well-attached to the stems. Fresh grapes have flexible green stems.	
Rotten Grape	Rotten grapes are those that have become sticky, fungal, or wrinkled at the stem connection. A mushy texture, brown dis-coloration, and a slight vinegar smell are some of the characteristics of rotten grapes. Mold will eventually emerge in rotten grape.	
Fresh Guava	Guavas are oval in form when they are fresh. Fresh guava has a distinct aroma, identical to that of lemon rind. The outer skin might be harsh and bitter, or it can be delicate and pleasant. The skin can be any thickness and is normally green before maturity, but can be maroon, green, or yellow when ripe, depending on the species.	
Rotten Guava	Inside, rotten guava turns brown or black. The guava has probably gone rotten if the peel splits, falls easily, or rots away. In the imperfect stage, Physalopara psidii produces stem canker and Diplodia metalenses produce dry grave fruit rot. The infection targets the plant's primary branches and stem, fracturing the lesion. The afflicted branches wilt when the stem tissues are killed.	
Fresh Jujube	When Jujubes are immature, they are green. As they ripen, they turn yellow-green with red-brown patches, and the completely mature fruit is completely red. However, we may consume them at any stage of development, from yellow-green to fully red; the redder they are, the sweeter they will taste. They have an apple-like texture and are crisp and delicious.	
Rotten Jujube	Small, round reddish to black marks appeared first on rotten jujube, and as they grew larger, they became deep brown to red-brown. Internal tissues became deep brown, while the damaged parts' surface became sunken with thicker fruit peel.	
Fresh Pomegranate	The pomegranate is a deciduous shrub belonging to the Lythraceae family and the Punicoideae subfamily. The pomegranate has a thick crimson skin that varies in color from yellow to purple and can contain up to 600 seeds. Fresh pomegranates are fruits that are hard on the outside and feel weighty for their size, have no cracks or bruises, and have firm, blemish-free skin.	
Rotten Pomegranate	Alternaria fruit rot causes the pomegranate to decay. It's also known as black rot because it causes wounds and decay on the inside of the fruit. Mold is arguably the most obvious indicator of a rotten pomegranate. The pomegranate has turned bad if the seeds are dark in color and have soft and mushy regions.	
Fresh Apple	Fresh apple skin is often green, yellow, pink, red, or russeted however, there are numerous bi- or tri-colored varieties available. Fresh apples have a firm texture, brilliant skin, and a nice, delicious aroma. They will be free of bruises, soft patches, and discoloration. They are crunchy and juicy when we bite into them.	
Rotten Apple	Apples that have gone bad will be mushy and brown in appearance. On the outer surface of the decaying apple, mold or fungus will start to grow. It will be light gray and may resemble a powdery patch.	
Fresh Banana	A banana has a curved shape and a thick, sweet-tasting skin. Depending on ripeness, the hue should range from green to dark yellow with brownish flecks. A banana is a tropical fruit that is widely consumed around the world. Fresh bananas are those that are slightly but not excessively green, bright in color, full and plump, and have no depressed, wet, or black spots on the peel.	
Rotten Banana	In a process known as enzymatic browning, high levels of ethylene cause the yellow pigments in bananas to degrade into those distinctive brown marks. If the banana has a lot of brown or black areas inside the peel mildew appears, or if it has an awful odor, it's probably past its prime.	
Fresh Orange	A firm orange with a thin, smooth peel and no soft patches is fresh. The fruit is tiny, not brightly colored, and delicious, with a pale yellow-hued juice, especially in lemon rootstock fruits. It's possible that the fresh orange is seedless or it may contain multiple tiny seeds.	
Rotten Orange	The peel of an orange is the most evident sign of its rottenness. Rotten orange is mushy and unpleasant with a sour odor. The flesh of a rotting orange is squishy and discolored. It might have a green and white soft spot on it.	
Fresh Strawberry	Strawberries have a juicy texture and are soft, sweet, vivid red fruit. Fresh strawberries have a vivid red hue, a natural gloss, and green crowns that appear to be new. Strawberry seeds are tiny edible seeds that develop all over the top of fresh strawberries. It should have a firm consistency but not be crunchy. Overly ripe strawberries might be excessively soft.	
Rotten Strawberry	A moldy odor or feel defines a rotten strawberry. It has a rotten smell and a damaged appearance. If the color, flavor, or texture of a strawberry changes from its original tone, it can be categorized as rotten strawberry. If there are any stains, flaws, or other symptoms of degradation, the strawberry has already become rotten.	

## 2 Experimental Design, Materials and Methods

### 2.1 Camera Specification

All the images were taken with a Single-lens reflex digital camera (Nikon D5600), which has an effective pixel of 24.2 million and 23.5 × 15.6 mm CMOS sensor. The total pixel of this camera is 24.78 million.

### 2.2 Preprocessing

**Fig 1:**
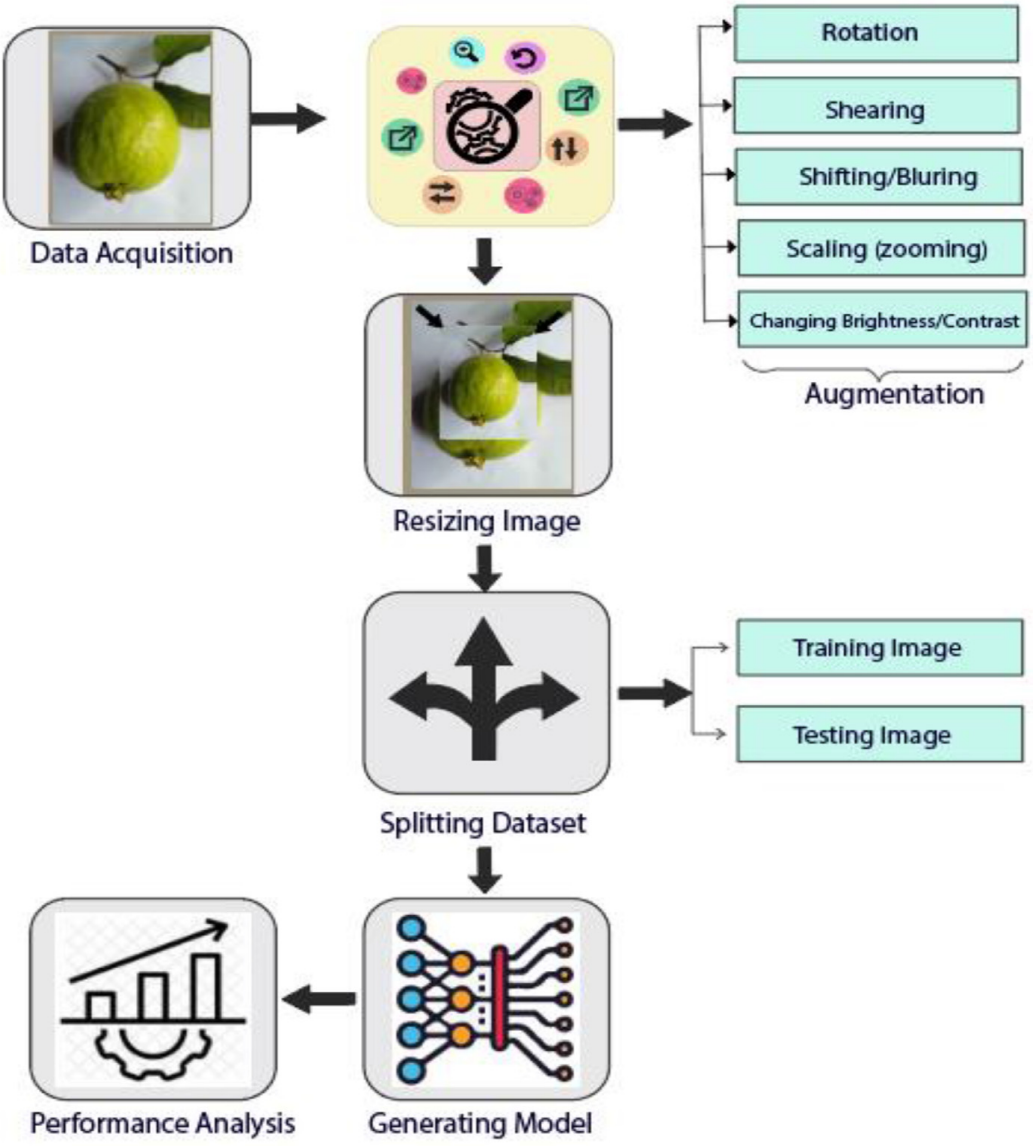
Working procedure of fresh and rotten fruit classification.

**Fig 2:**
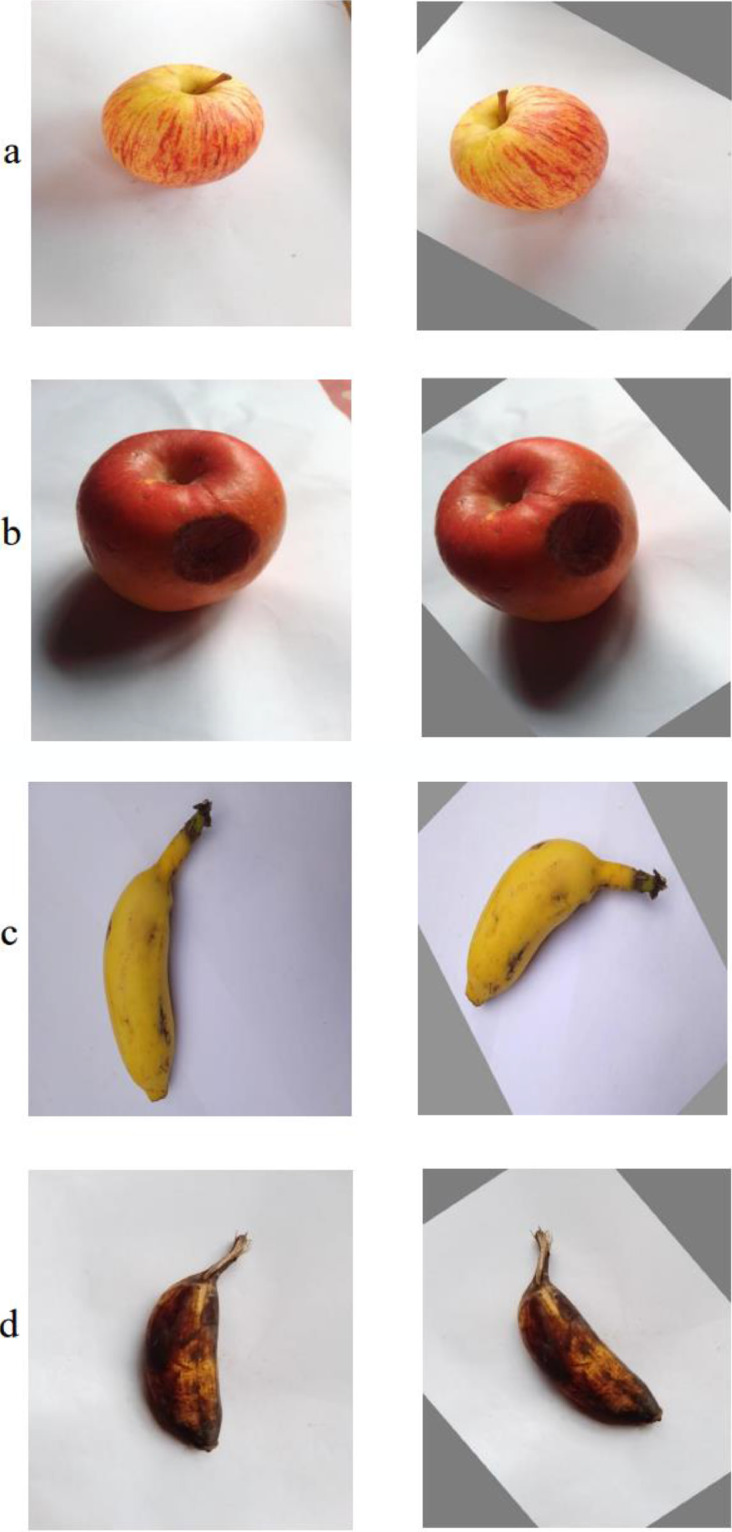

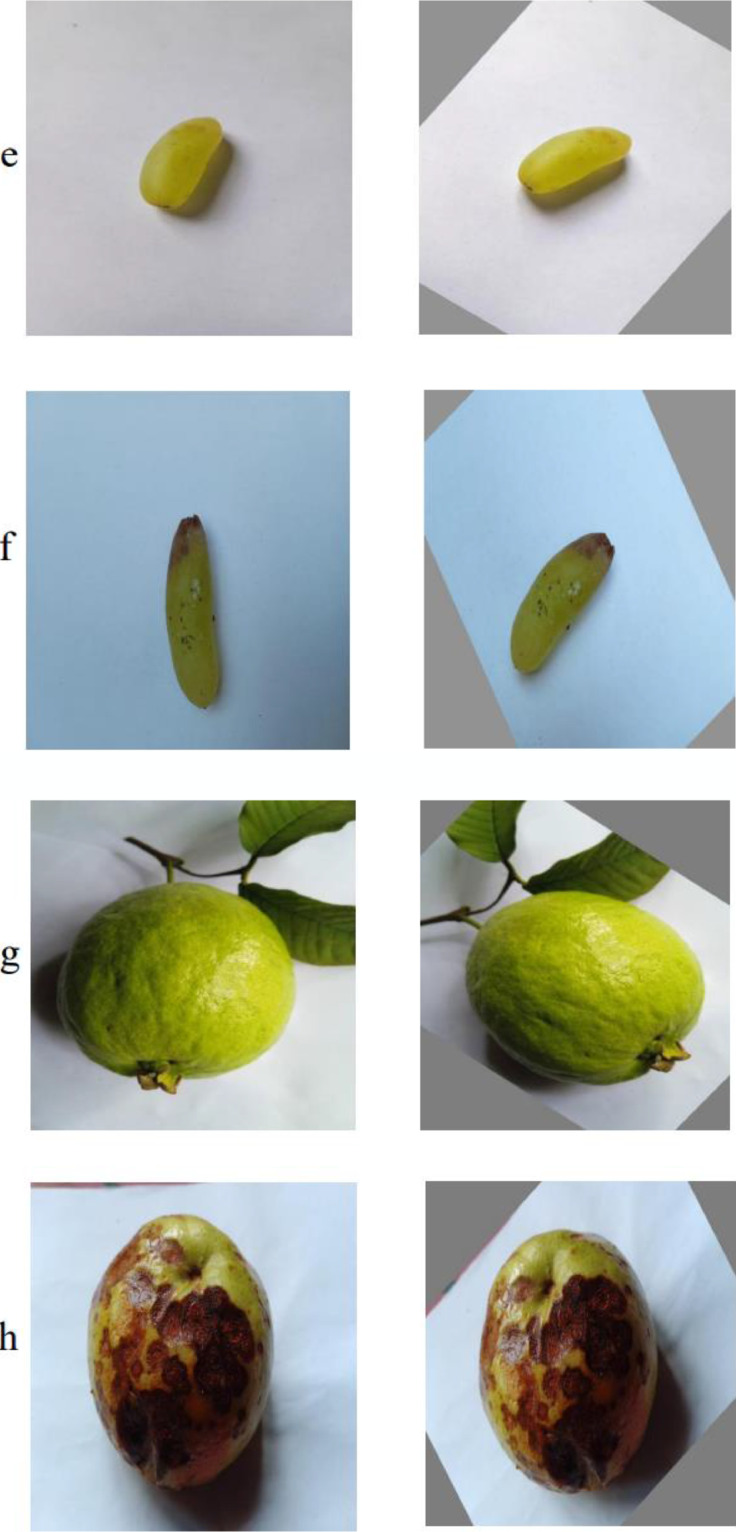

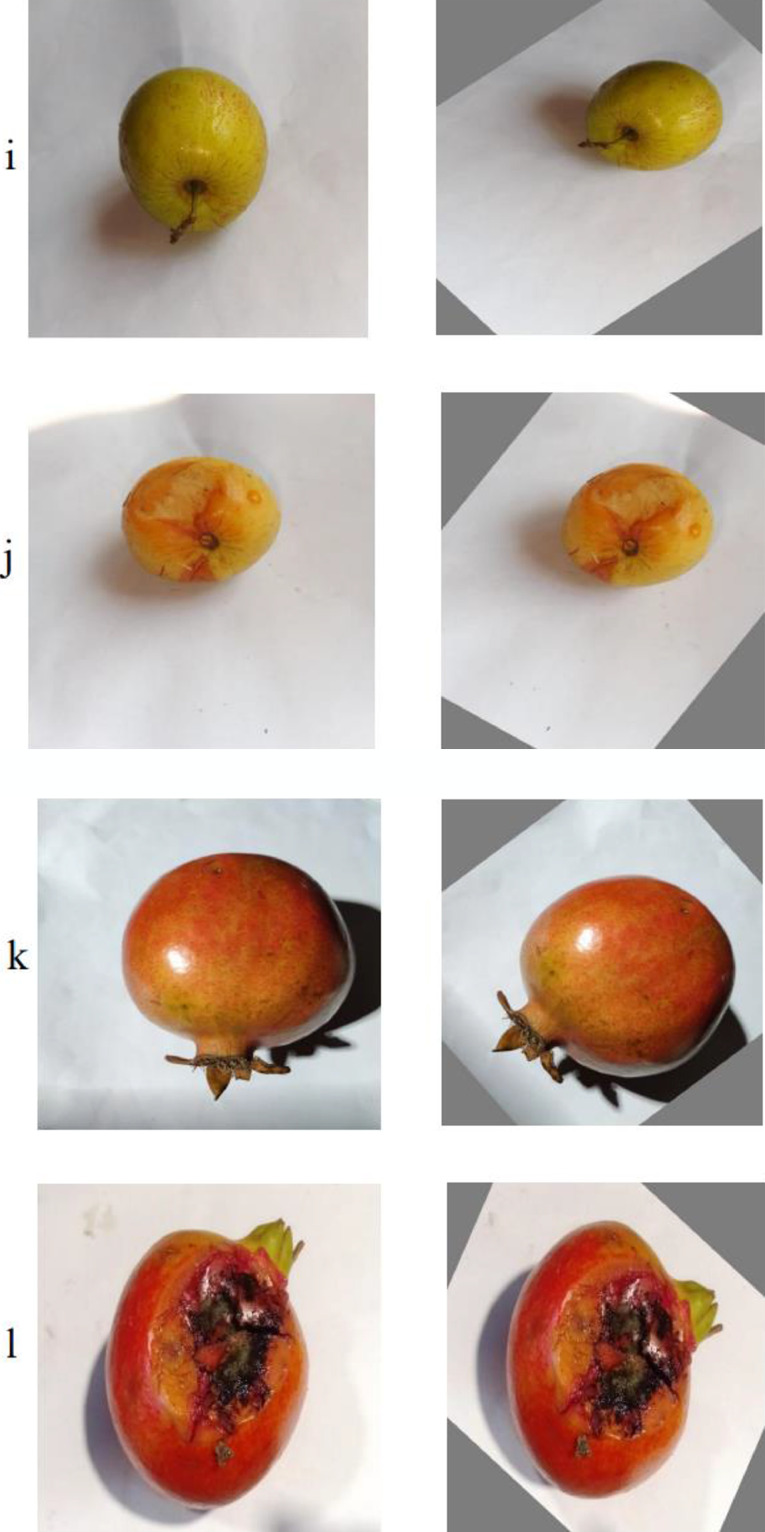

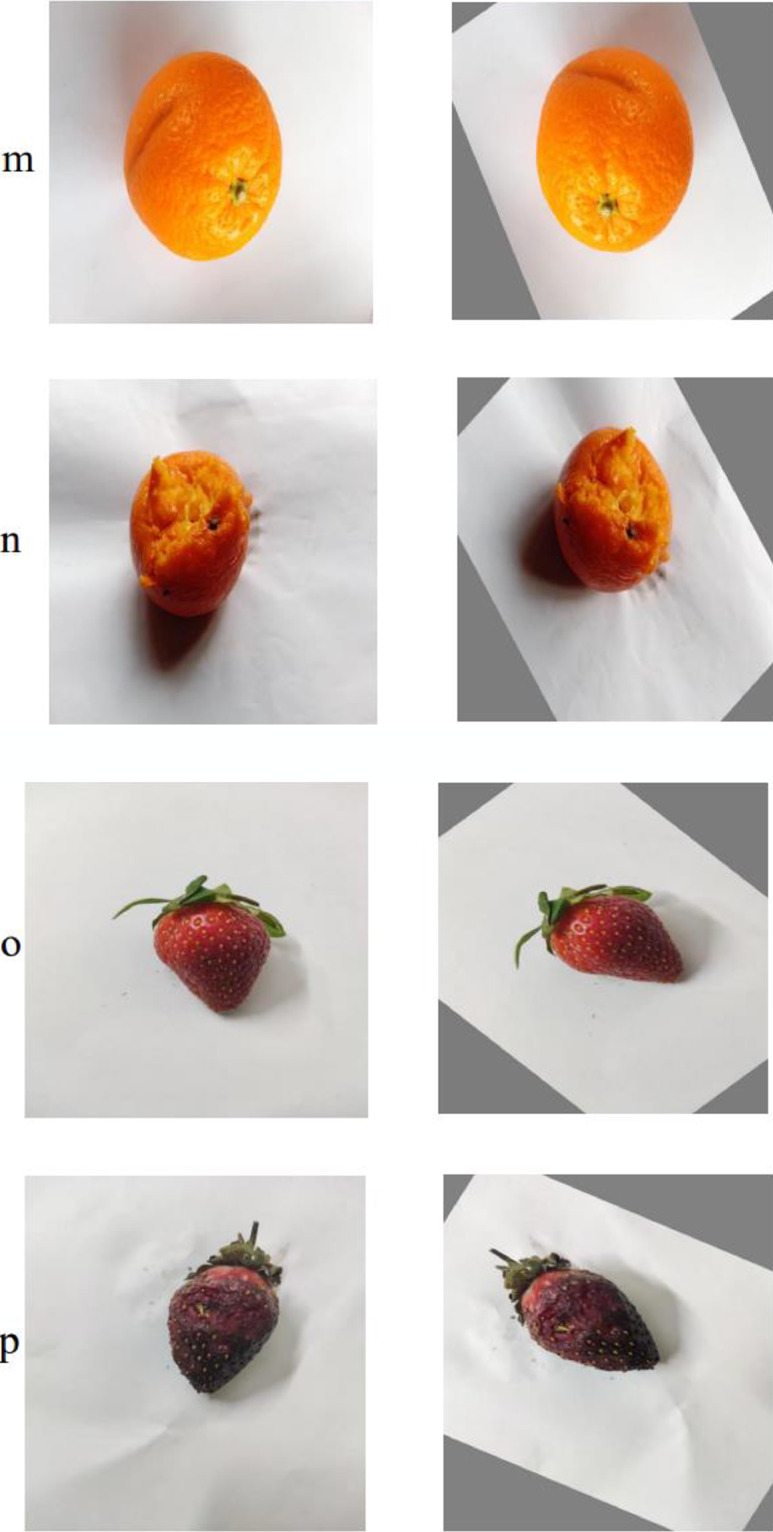
(i): Example of augmented images with (a) fresh apple (b) rotten apple. (ii): Example of augmented images with (a) fresh banana (b) rotten banana, (iii): Example of augmented images with (a) fresh grape (b) rotten grape, (iv): Example of augmented images with (a) fresh grape (b) rotten grape, (v): Example of augmented images with (a) fresh guava (b) rotten guava, (vi): Example of augmented images with (a) fresh pomegranate (b) rotten pomegranate, (vii): Example of augmented images with (a) fresh orange (b) rotten orange, (viii): Example of augmented images with (a) fresh strawberry (b) rotten strawberry

For training our dataset using any deep learning model, we have to perform five steps named (a) Augmentation, (b) Resizing images, (c) Splitting dataset, (d) Generating Model, (e) Performance Analysis. We visualized these steps in Fig 1. Since we require a vast amount of data for training the deep learning model, we increased our data using augmentation. Augmentation was done through Random rotation, scaling, shearing, cropping, shifting, adjusting brightness, contrast, saturation, and hue. Some Sample augmented images for this fruit dataset are given in Fig. 2. We performed rotation with 45°, 60°, and 90° angles. Some techniques that we applied for re-scaling are Nearest-Neighbor Interpolation, Bicubic Interpolation, and Bilinear Interpolation. Histogram Equalization is another approach that we used to improve image contrast. The value of the width shift range, height shift range, and sheer range is 0.2 used for augmentation [4]. The value of the width shift range, height shift range, and sheer range is 0.2 used for augmentation [4]. We used the fill mode() function from the Keras package in Python for estimating parameters. Before being fed into the neural network model, we have resized all the images in a fixed size and shape. We used 512 × 512 pixels to resize the images. Then we divided resized images into training and testing sets. In the future, we will investigate the deep learning models in detail using this dataset to find an optimum method for real-life applications.

## Ethical Approval (involvement of animals)

This article does not contain any studies with animals performed by any of the authors.

## Ethical Approval (involvement of human subjects)

There are no studies involving human participants done by any of the authors in this article. The datasets used in the article are open to the public. For the usage of these datasets, proper citation rules should be maintained.

## CRediT authorship contribution statement

**Nusrat Sultana:** Conceptualization, Writing – original draft, Methodology, Software, Data curation, Visualization. **Musfika Jahan:** Conceptualization, Writing – original draft, Methodology, Software, Data curation, Visualization. **Mohammad Shorif Uddin:** Supervision, Writing – review & editing.

## Declaration of Competing Interest

The authors declare that they have no conflict of interests from any competing financial interests or personal relationships that could have appeared to influence the work reported in this paper.

## Data Availability

Fresh and Rotten Fruits Dataset for Machine-Based Evaluation of Fruit Quality (Original data) (Mendeley Data).
